# Supplementary material for: Conditional chemoconnectomics (cCCTomics) as a strategy for efficient and conditional targeting of chemical transmission
Source: eLife. 2024 Apr 30;12:RP91927. doi: 10.7554/eLife.91927 (PMC11060718; doi:10.7554/eLife.91927)
Supplement: Figure 3—source data 3. [file elife-91927-fig3-data3.zip › Figure 3 source data3 part 2.zip!/Figure 3-Source data 3-part2/1060f2-1-CRISPResso_Report_gl5umwyq.zip!/CRISPResso_on_1060f2_4/9.Alleles_frequency_table_around_sgRNA_gcccacttcctggagtatcg.pdf]

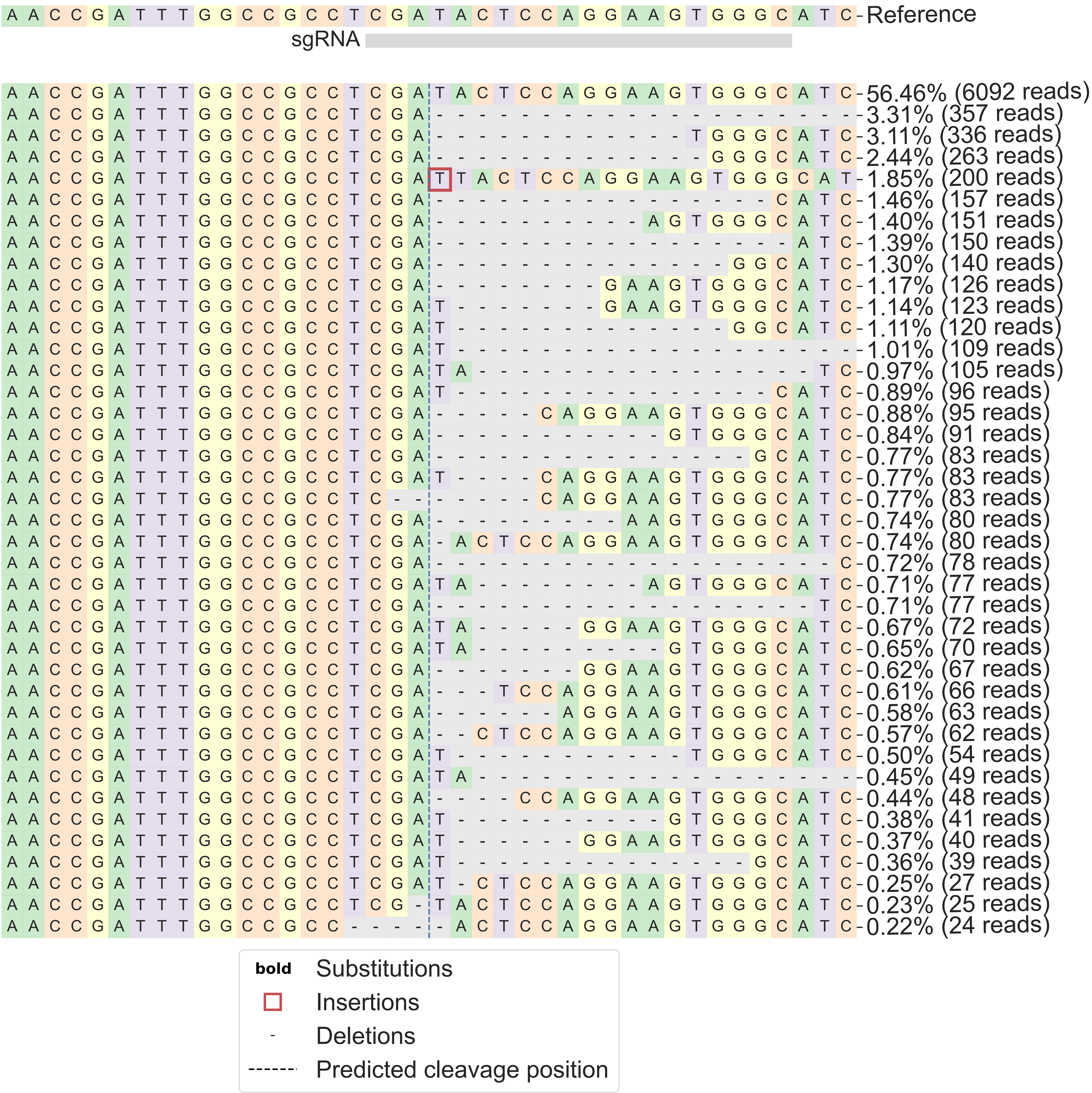

AACCGATTTGGCCGCCTCGATACTCCAGGAAGTGGGCATC
Reference
sgRNA
56.46% (6092 reads)
3.31% (357 reads)
3.11% (336 reads)
2.44% (263 reads)
1.85% (200 reads)
1.46% (157 reads)
1.40% (151 reads)
1.39% (150 reads)
1.30% (140 reads)
1.17% (126 reads)
1.14% (123 reads)
1.11% (120 reads)
1.01% (109 reads)
0.97% (105 reads)
0.89% (96 reads)
0.88% (95 reads)
0.84% (91 reads)
0.77% (83 reads)
0.77% (83 reads)
0.77% (83 reads)
0.74% (80 reads)
0.74% (80 reads)
0.72% (78 reads)
0.71% (77 reads)
0.71% (77 reads)
0.67% (72 reads)
0.65% (70 reads)
0.62% (67 reads)
0.61% (66 reads)
0.58% (63 reads)
0.57% (62 reads)
0.50% (54 reads)
0.45% (49 reads)
0.44% (48 reads)
0.38% (41 reads)
0.37% (40 reads)
0.36% (39 reads)
0.25% (27 reads)
0.23% (25 reads)
0.22% (24 reads)
bold Substitutions
Insertions
Deletions
Predicted cleavage position
